# Postprandial Hypotension—Methods for the Evaluation and Management

**DOI:** 10.1111/ggi.70456

**Published:** 2026-04-01

**Authors:** Joji Ishikawa, Ayumi Toba, Shutaro Futami, Kazumasa Harada

**Affiliations:** ^1^ Department of Cardiology Tokyo Metropolitan Institute for Geriatrics and Gerontology Tokyo Japan

**Keywords:** ambulatory blood pressure monitoring, home blood pressure monitoring, orthostatic hypotension, postprandial hypotension, supine hypertension

## Abstract

Postprandial hypotension (PPH) is a common but underrecognized condition in older adults that affects nearly half of the population and is frequently associated with autonomic dysfunction. Unlike orthostatic hypotension (OH), PPH often presents with sleepiness and syncope. Its prevalence and severity are influenced by meal composition, particularly carbohydrate content, and the time of day, with morning meals posing a greater risk. Its pathophysiology involves splanchnic blood pooling, altered gastric emptying, impaired baroreflex function, and intestinal peptide activity, including those of glucagon‐like peptide (GLP)‐1 and GLP‐2. Despite its clinical significance, PPH lacks standardized diagnostic criteria. Conventional blood pressure (BP) measurements, ambulatory BP monitoring (ABPM), and home BP monitoring (HBPM) have been used to detect PPH, with ABPM offering continuous data and HBPM providing practical position‐controlled assessments. However, variability in test meals and measurement protocols limits consistency across studies. Management primarily involves lifestyle modifications, such as premeal water intake, smaller and low‐carbohydrate meals, and postprandial exercise. Pharmacological interventions, including acarbose, which targets PPH associated with autonomic dysfunction, may be considered in select cases. Emerging therapies involving gut peptides, such as GLP‐1 analogs and DPP‐4 inhibitors, show promise, especially in patients with α‐synucleinopathies. PPH frequently coexists with OH and supine hypertension, complicating diagnosis and treatment. Screening for positional BP dysregulation and tailoring interventions for individual autonomic profiles are essential. Given its association with cardiovascular events and mortality, the improved recognition and management of PPH are critical in geriatric care.

## Graphical abstract

Postprandial hypotension (PPH) is highly prevalent in older patients; however, its diagnosis remains challenging due to the absence of a consensus guideline for diagnostic criteria. Lifestyle modification is the first‐line management, taking into account the pathophysiology underlying PPH and the coexisting conditions of orthostatic hypotension and supine hypertension.

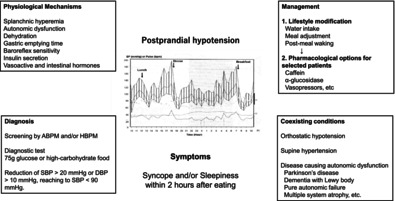

## 1 Introduction

Postprandial hypotension (PPH) may occur in patients with autonomic dysfunction, even in the absence of neurological symptoms associated with Parkinson's disease (PD) or dementia with Lewy bodies (DLB), and often leads to sleepiness and syncope. Although PPH is more prevalent than orthostatic hypotension (OH), its detection and clinical understanding remain challenging due to the lack of a standardized definition or consensus guidelines. This narrative review examines the current diagnostic approaches for PPH, including traditional blood pressure (BP) measurement, ambulatory BP monitoring (ABPM), and home BP monitoring (HBPM). It also discusses the prevalence, underlying mechanisms, and management strategies.

## 2 Methods

This review is based on articles identified through a PubMed search using the term “postprandial hypotension.” We selected potentially relevant articles based on their titles and abstracts, and the first author reviewed the full texts to determine which studies to include in this narrative review.

### 2.1 Prevalence and Symptoms of PPH

According to a meta‐analysis, PPH has been reported in 40.5% of older adults, with the prevalence varying by setting: 32.8% in community‐dwelling individuals, 39.4% in long‐term care facilities, and 49.3% in hospital geriatrics departments [1]. In a 75 g glucose‐loading study, 19.9% of the general population exhibited a PPH [2]. Patients with autonomic dysfunction showed a significantly higher prevalence (3.49‐fold in PD, 6.61‐fold in Alzheimer's disease, and 4.83‐fold in diabetic neuropathy) than healthy controls [3]. Moreover, peripheral autonomic failure is associated with more severe PPH than central autonomic failure [4].

Approximately two‐thirds of the patients with PPH present with symptoms, most commonly sleepiness and syncope [5]. OH typically manifests as dizziness and falls [5]. OH and PPH do not necessarily co‐occur in geriatric patients, suggesting that they have distinct pathophysiological mechanisms [6]. PPH is more common than OH in older adults with DLB [7].

Beyond its immediate symptoms, PPH has been linked to serious outcomes, including falls and syncope [8], dizziness, postural instability, coronary events, stroke [9, 10], and increased all‐cause mortality during long‐term follow‐up [11, 12]. A meta‐analysis confirmed that individuals with PPH are at an elevated risk of cardiovascular diseases, stroke, and mortality [13]. PPH has also been shown to predict all‐cause mortality even among older adults receiving low‐level care [14]. Furthermore, in a prospective cohort study with 36‐month follow‐up, PPH was identified as a risk factor for the development of new cardiovascular disease in community‐dwelling older individuals [15]. In older patients with hypertension, marked PPH indicates advanced cerebrovascular damage even in the absence of overt neurological findings [9].

### 2.2 Mechanisms Underlying PPH

**FIGURE 1:**
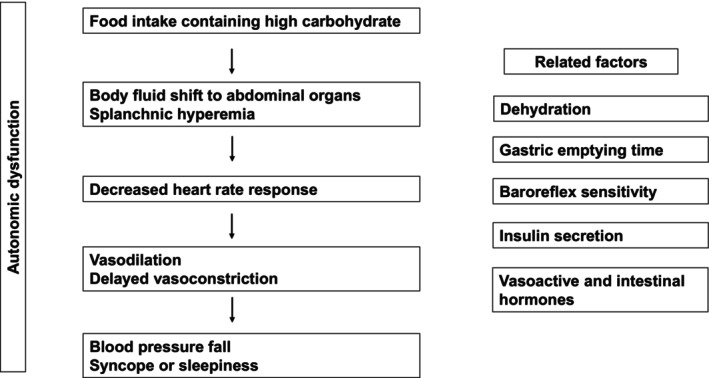
Physiological mechanisms associated with postprandial hypotension.

Multiple physiological mechanisms have been implicated in the pathogenesis of PPH (Table 1) and are shown in Figure 1. In patients with autonomic nerve dysfunction, contributing factors may include intake of high‐carbohydrate meals, postprandial pooling of visceral blood, abnormal gastric emptying, impaired baroreflex function, and altered secretion of gastrointestinal hormones.

**TABLE 1:** Possible mechanisms of postprandial hypotension (PPH).

Mechanism/factor	Key findings/evidence
High‐carbohydrate meals [16]	Larger SBP drop (−40 mmHg) and longer duration vs. low‐carbohydrate meal (−28 mmHg); more symptoms.
Postprandial splanchnic hyperemia [17]	Increased mesenteric blood flow → ↓SBP/DBP, ↑HR, ↑glucose.
Abnormal gastric emptying [18, 19]	Mixed findings: rapid emptying linked to PPH [18]; slower emptying also associated with greater BP drop [19].
Impaired baroreflex/sympathetic response [6]	Reduced baroreflex effectiveness postmeal; inadequate cardiac output and compensation [6].
Intestinal peptides [20, 21]	↑GLP‐1, ↓GLP‐2 secretion → visceral pooling; insulin/glucose effects inconsistent.

Abbreviations: DBP, diastolic blood pressure; GLP, glucagon‐like protein; SBP, systolic blood pressure.

High‐carbohydrate meals influence the magnitude, duration, and symptoms of PPH [16]. The maximum decrease in systolic BP (SBP) was significantly smaller after a low‐carbohydrate meal (25 g; −28 ± 5 mmHg) compared to normal (65 g; −39 ± 7 mmHg) and high‐carbohydrate meals (125 g; −40 ± 5 mmHg). Additionally, the duration of hypotension and severity of postprandial symptoms were significantly reduced for the low‐carbohydrate meals.

Postprandial splanchnic hyperemia has also been identified as a key contributor to PPH [8, 22]. After meals, patients exhibit a significant reduction in SBP and diastolic BP (DBP), elevated heart rate, and increased postprandial glucose levels, all of which are associated with enhanced blood flow in the superior mesenteric artery compared to the fasting state [17].

Abnormal gastric emptying has also been proposed as another mechanism, though findings are inconsistent. One study found that PPH was associated with accelerated gastric emptying in healthy older adults [18], while another longitudinal study (~5.8 years) reported that PPH prevalence increased alongside modest gastric emptying delays, with slower emptying correlated with greater hypotensive response [19].

Impaired baroreflex function, inadequate postprandial increases in cardiac output, and insufficient sympathetic compensation are also considered major contributors [6]. In meal testing, patients with PPH exhibited an acute postprandial reduction in baroreflex effectiveness compared to controls [23]; however, other studies have reported no significant differences in baroreflex sensitivity between individuals with and without PPH [24].

Vasoactive and intestinal peptides may further influence PPH pathophysiology [20]. While PPH has been linked to exaggerated postprandial insulin secretion [25], studies in healthy individuals have not found consistent associations with insulin or glucose levels [26], suggesting that additional mechanisms are involved. The small intestine plays a central role in nutrient‐gut interactions and neurohormonal responses, including the secretion of glucagon‐like peptide‐1 (GLP‐1), glucose‐dependent insulinotropic peptide (GIP), and somatostatin, all of which influence postprandial hemodynamics [21]. Fukushima et al. [20] reported that patients with multiple system atrophy and PPH exhibited higher postprandial GLP‐1 levels and reduced GLP‐2 secretion than those without PPH, which contributed to increased intestinal blood pooling.

### 2.3 Diagnosis Approaches in PPH

Definitions, test meals, timing of BP measurements, and time‐of‐day effects in the diagnosis of PPH are summarized in Table 2.

**TABLE 2:** Diagnostic approaches in postprandial hypotension (PPH).

Method/observation	Protocol/conditions	Key findings/criteria	Notes
Traditional definition of PPH [6]	SBP drop > 20 or < 90 mmHg (if baseline > 100 mmHg) within 2 h postmeal	—	No international consensus guideline
Simplified diagnostic method by Abbas et al. [27]	SBP drop ≥ 10 mmHg between premeal and 75 min postmeal	Sensitivity: 82%, specificity: 91%	Good reproducibility (ICC 0.88)
Test meal or liquid			
OGTT in general population [2]	75 g glucose drink; BP and HR measured at 1 h and 2 h	SBP: −6.2/−8.1 mmHg, DBP: −4.7/−6.1 mmHg, HR: +4.3/+2.6 bpm	BP nadir in 30–60 min (70%); delayed drop at 75 min (15%)
Meal contains 65 g of carbohydrate [16]	SBP drop > 20 or < 90 mmHg (if baseline > 100 mmHg) within 2 h postmeal	SBP drop by 65 g carbohydrate: −39 ± 7 mmHg	SBP drop by low‐carbohydrate meal (25 g), (−28 ± 5 mmHg): by high‐carbohydrate meals (125 g), (−40 ± 5 mmHg)
Measurement timing [1]	Start: 5–45 min postmeal; duration: 30–120 min	—	Timing affects diagnostic accuracy
Time‐of‐day effects [28]	Comparison across breakfast, lunch, and dinner	Most prevalent in the morning; shortest duration and mildest symptoms after dinner	PPH often occurs after breakfast in older adults with falls/syncope

In a study of the general population [2], SBP was decreased by SBP and DBP by 6.2 and 8.1 mmHg, and by 4.7 and 6.1 mmHg, respectively, while heart rate increased by 4.3 and 2.6 bpm, at 1 and 2 h after ingestion of a 75 g glucose drink. Postprandial BP typically reaches its nadir within 30–60 min in approximately 70% of the patients. However, in approximately 15% of patients, a significant decrease in SBP is still observed as late as 75 min after a meal [6]. The reported time intervals for postprandial BP measurements range from 5 to 45 min, with durations spanning 30–120 min [1].

PPH is most prevalent in the morning and least prevalent in the evening. Furthermore, test meals administered at dinnertime induce significantly shorter hypotensive periods, with patients experiencing fewer or no symptoms than those tested at breakfast or lunchtime [28]. Approximately one in four older patients with a history of falls or syncope experience PPH, most commonly after breakfast [29]. Therefore, diagnostic testing should be conducted under conditions that closely resemble those of symptom onset, including meal timing and medication use [30].

Traditionally, PPH has been defined as a fall in SBP > 20 mmHg or a decrease to < 90 mmHg when the preprandial SBP exceeds 100 mmHg, occurring within 2 h after a meal [6]. However, there are currently no consensus guidelines for the diagnosis of PPH. A simplified diagnostic method has been proposed [27], defining PPH as a decrease of at least 10 mmHg in SBP between premeal and 75 min postmeal measurements, with a reported sensitivity of 82% and specificity of 91%. The reproducibility of PPH diagnosis is considered good: SBP declined by an average of 16 ± 4 and 12 ± 4 mmHg during the first and second meal studies, respectively, with an intra‐class correlation coefficient of 0.88 (95% CI: 0.85–0.97) [31].

Due to the absence of standardized guidelines for test meals for PPH diagnosis, meal composition varied across studies [1]. Some studies employed meals containing 65 g of carbohydrates [5, 16], while others used a 75 g glucose drink [2].

### 2.4 PPH in ABPM

Diagnostic approaches for PPH using ABPM and HBPM are summarized in Table 3.

**TABLE 3:** Diagnostic approaches in postprandial hypotension (PPH) using ambulatory blood pressure monitoring (ABPM) or home blood pressure monitoring (HBPM).

Method	Protocol/conditions	Key findings/criteria	Notes
ABPM (standardized meal) [5, 9, 32]	65 g Carbohydrate liquid meal	Effective for PPH detection	Recommended: 10–30 min intervals up to 2 h [33]
ABPM (conventional 24‐h) [34]	Every 30 min without fixed meals	PPH ≥ 20 mmHg drop in mean SBP within 2 h postmeal	The within‐subject reproducibility was low [34]
PPH predictors (ABPM) [35]	Daytime SBP standard deviation > 10 mmHg	Sensitivity: 87%, Specificity: 57%	Also predicts autonomic dysfunction (OR 3.75) [36]
HBPM (KAMOGAWA‐HBP study) [37]	6 readings per meal (pre, immediately post, 30, 60, 90, 120 min) over 3 days	50% of diabetic outpatients had PPH	High premeal SBP, HbA1c, autonomic dysfunction = risk factors
HBPM (Barochiner et al.) [38]	Duplicate readings for 4 days: morning, 1 h before/after lunch, evening	PPH prevalence: 27.4%; age > 80, low BMI, high office SBP, cerebrovascular history associated	Practical screening tool for older patients who are hypertensive
HBPM (Alfie et al.) [39]	Before/after 3 consecutive lunches	PPH prevalence: 27.4%; 13.2% in controlled HT, 42.2% in uncontrolled HT	

Vloet et al. [5] employed a validated ABPM device to assess PPH. During the evaluation, patients rested in a seated position for 20 min before ingesting a standardized liquid test meal within 10 min. The meal consisted of 100 mL of glucose syrup and 100 mL of lactose‐free whole milk, providing 65 g of carbohydrates, 2 g of fat, and 4 g of protein. SBP, DBP, and heart rate were recorded every 10 min from 20 to 90 min after the start of meals. The diagnostic methodology involved measuring BP at intervals of 10–30 min for up to 2 h postmeal [33].

Kohara et al. [9, 32] used 24‐h ABPM to evaluate PPH in hospitalized patients with hypertension. BP was measured every 30 min from 6:00 a.m. to 10:00 p.m., and every 60 min from 10:00 p.m. to 6:00 a.m. the following day. The meal content, timing, and daily activities were standardized. The total caloric intake was set at 30 kcal/kg of ideal body weight per day, with a macronutrient composition of 66% carbohydrates, 16% protein, and 18% fat. Meals were served at 8:00–8:30 a.m., 12:00–12:30 p.m., and 6:00–6:30 p.m. The postprandial BP change was defined as the difference between the mean SBP 1 h before and 2 h after each meal. However, averaging SBP over a 2‐h period may underestimate the true decline, and measurements every 10 min during this window are recommended [11].

**FIGURE 2:**
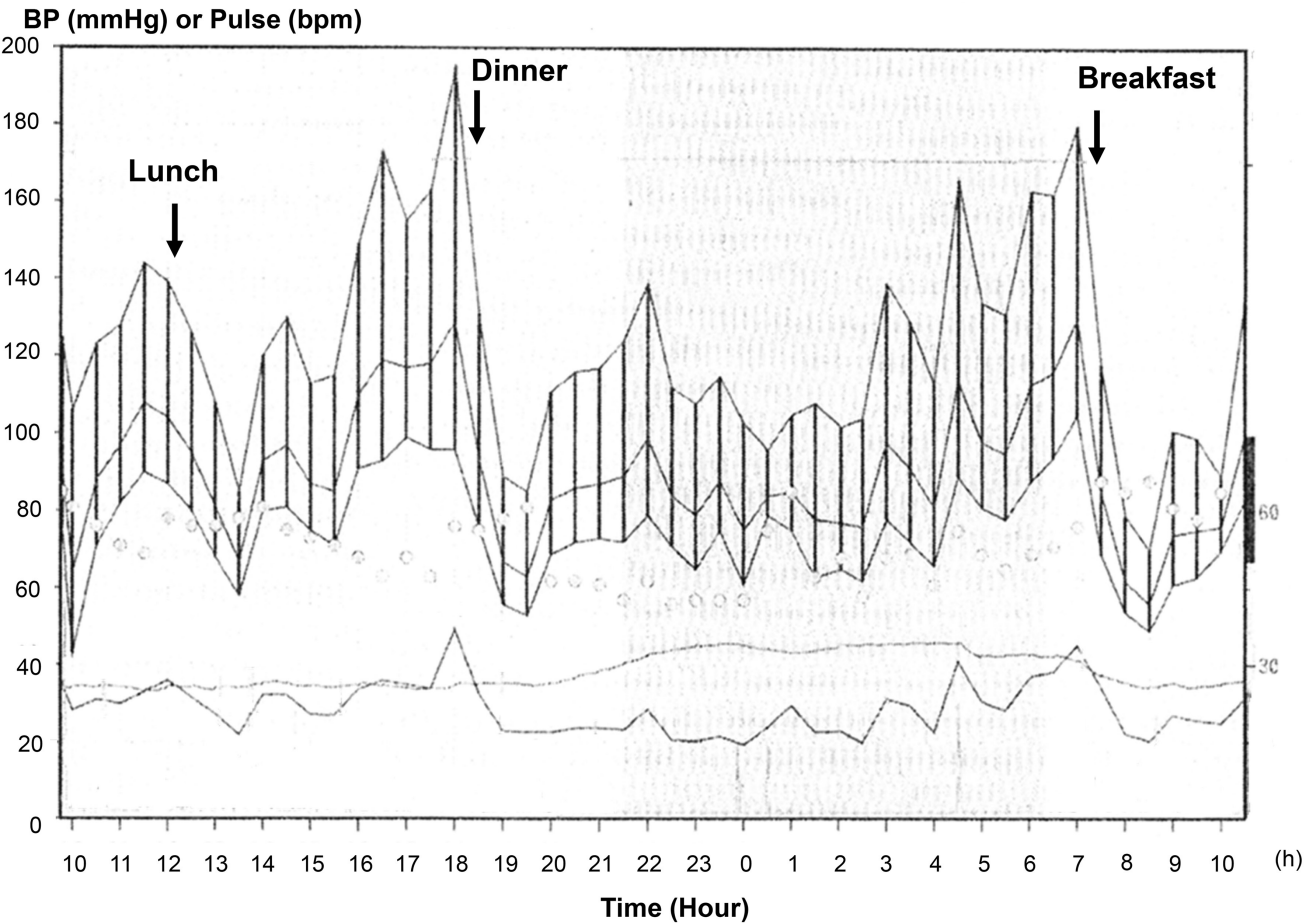
Typical ABPM in a patient with PPH associated with Parkinson's disease.

Most ABPM‐based diagnoses of PPH rely on a single standardized test conducted at fixed meal times [5, 29, 40]. Typical ABPM in a patient with PPH associated with Parkinson's disease is shown in Figure 2. In conventional 24‐h ABPM, Grodzicki et al. [34], as part of the Systolic Hypertension in Europe (SYST‐EUR) Trial, defined PPH as a ≥ 20 mmHg decline in average SBP during the 2 h following a meal. While group‐level reproducibility was acceptable, within‐subject reproducibility was low [34], likely because of variations in physical activity and posture after meals. Thus, the applicability of conventional 24‐h ABPM in patients with hypertension without standardized conditions for PPH diagnosis remains uncertain.

A postprandial BP decline contributes to overall BP variability. Delta and preprandial SBP were positively correlated [29]. A standard deviation of SBP > 10 mmHg was predictive of PPH, with a sensitivity and specificity of 87% and 57%, respectively [35]. Moreover, PPH and heart rate variability measured via conventional 24‐h ABPM were strong predictors of autonomic dysfunction in routine clinical settings [36], with PPH associated with a nearly fourfold increased risk (odds ratio [OR], 3.75) [36].

### 2.5 Detection of PPH in HBPM

HBPM allows repeated measurements in a seated position without the confounding effects of physical activity or postural changes. Therefore, HBPM use for PPH diagnosis has recently been reported.

In the KAMOGAWA‐HBP study [37], patients with diabetes were instructed to perform three sets of six BP measurements around their main meal of the day: before the meal, immediately after, and at 30, 60, 90, and 120 min postprandially. Approximately half of the outpatients with diabetes were found to have PPH. Higher preprandial SBP was significantly associated with an increased PPH risk. Older adults and patients with elevated hemoglobin A1c levels or autonomic dysfunction have difficulty recognizing PPH symptoms. However, the practicality of this method, which requires 18 BP readings during a single meal, may be limited in routine clinical settings.

Barochiner et al. [38] conducted HBPM over 4 days, with duplicate measurements taken in the morning, 1 h before and after lunch, and in the evening, to assess PPH in older patients with hypertension. They reported a PPH prevalence (defined as ≥ 1 episode) of 27.4%. Factors associated with PPH included age > 80 years (OR, 3.45), low body mass index (OR, 0.88), high office SBP (OR, 1.03), and history of cerebrovascular disease (OR, 3.29).

Alfie et al. [39] performed HBPM before and after three consecutive lunches and found that unsuspected PPH occurred in 27.4% of patients with hypertension. The prevalence of PPH was 13.2% in patients with controlled hypertension and 42.2% in those with uncontrolled hypertension.

Since antihypertensive medications are typically taken after breakfast, PPH frequently occurs in the morning; evaluating PPH before and after lunch or dinner may help avoid the confounding effects of medication. Nonetheless, the safety assessment of morning antihypertensive administration remains important, particularly in patients with PPH. Notably, PPH after breakfast was positively associated with the morning BP surge observed in the ABPM [32], highlighting the challenge of achieving intensive BP control based on home BP measurements taken immediately after waking and before breakfast.

**FIGURE 3:**
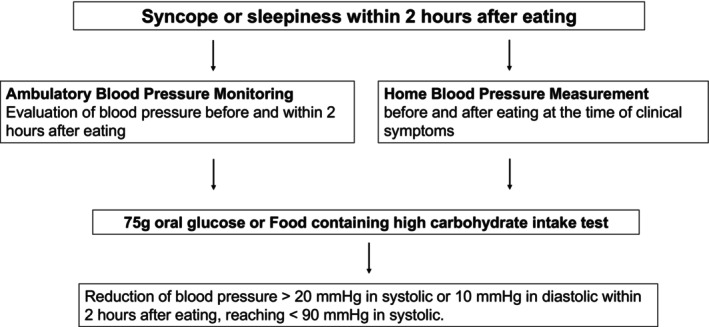
Flowchart of diagnosis of PPH using ABPM and/or HBPM.

Possible flowchart of diagnosis of PPH using ABPM and/or HBPM is shown in Figure 3.

### 2.6 Management Strategies for PPH

Management strategies for PPH and coexisting positional BP dysregulation are summarized in Table 4. Lifestyle modification is the first‐line therapy for PPH, followed by pharmacological options for selected patients.

**TABLE 4:** Management strategies for postprandial hypotension (PPH) and coexisting positional BP dysregulation.

Category	Intervention/strategy	Details/notes
Lifestyle modifications	Water intake [41]	500 mL before meals reduces PPH in older adults
	Meal adjustment [11, 16, 22]	Smaller, low‐carb meals; increased meal frequency
	Postmeal walking [42]	Mild aerobic walking ~10 min, starting 20 min after meals; improves BP, but the effect is not sustained after stopping
Pharmacologic therapies	Acarbose [43, 44]/voglibose [45]	α‐glucosidase inhibitors; delay gastric emptying, reduce SBP drop, HR rise, and postprandial glucose; effective in diabetes and autonomic failure
	Somatostatin analogs [46]	Octreotide reduces splanchnic blood pooling
	Caffeine/cold glucose [47]	May acutely raise BP
	NSAIDs/CGRP [46]	Potential vasoconstrictive effects
	DPP‐4 inhibitors [48, 49]	Vildagliptin, sitagliptin: case reports suggest benefit
	GLP‐1 analogs [21]	Potential future therapy for PPH with autonomic dysfunction (e.g., Parkinson's)
	Norepinephrine‐based therapy [50]	Replacers (midodrine, droxidopa) for low sympathetic reserve; enhancers (pyridostigmine, atomoxetine, yohimbine) for preserved reserve
Coexisting OH (orthostatic hypotension)	Screening [51]	Measure BP/HR supine and after 3 min standing; SBP drop ≥ 20 mmHg or DBP ≥ 10 mmHg
	Patient education [51]	Rise slowly, avoid heat/alcohol/large meals, use physical counter‐maneuvers
Supine hypertension	Definition [52]	SBP ≥ 140 mmHg or DBP ≥ 90 mmHg after > 5 min supine
	Non‐pharmacologic management	Head‐up tilt during sleep, small bedtime snack, nighttime enteral feeding

Lifestyle modification is the cornerstone of PPH management. Several non‐pharmacological and pharmacological interventions have been proposed, including delayed gastric emptying with moderate gastric distension, premeal water ingestion, mild‐intensity postprandial walking (initiated approximately 20 min after breakfast), cold glucose loading, caffeine intake [47], somatostatin analogs (e.g., octreotide), nonsteroidal anti‐inflammatory drugs (NSAIDs), calcitonin gene‐related peptide, and α‐glucosidase inhibitors such as acarbose [46].

Drinking a large volume (500 mL) of water before meals attenuates PPH in older adults [41]. Reducing meal size [11] and lowering carbohydrate content [16, 22] can also reduce the magnitude and duration of PPH. Consequently, smaller and more frequent meals represent a simple, cost‐effective, and effective intervention for older patients [11].

Postprandial walking may alleviate PPH‐related symptoms because frail older adults with PPH exhibit increased postprandial BP and heart rate during walking. However, this effect is not sustained once walking ceases; therefore, patients with symptomatic PPH should either continue walking or remain seated [42].

Vasoactive agents should be used cautiously and only under specific conditions because patients with autonomic dysfunction often exhibit exaggerated BP variability, transient hypertensive episodes, and supine hypertension. Treatment with levodopa/benserazide (125 mg, twice daily) did not significantly improve orthostatic or PPH in patients with PD [53]. Understanding the underlying pathophysiology is essential for selecting appropriate therapies [50]. For example, patients with low “sympathetic reserve” (e.g., those with peripheral noradrenergic degeneration such as pure autonomic failure or PD) and low plasma norepinephrine levels tend to respond better to norepinephrine replacers (e.g., midodrine, droxidopa). In contrast, patients with relatively preserved sympathetic reserves (e.g., those with central autonomic impairment, such as multiple system atrophy) may respond better to norepinephrine enhancers (e.g., pyridostigmine, atomoxetine, and yohimbine) [50].

Acarbose, an α‐glucosidase inhibitor, is effective in reducing the severity and symptoms of PPH [43]. Shibao et al. reported that 100 mg of acarbose improved PPH in patients with severe autonomic failure, an effect that is not solely attributable to reduced insulin levels [44]. Acarbose significantly attenuated postprandial SBP decline, heart rate increase, blood glucose elevation, and superior mesenteric artery blood flow [17]. It also reduces BP fluctuations in older patients with diabetes [54]. Similar results were obtained for voglibose [45].

PPH has been linked to GLP‐1 and decreases the postprandial secretion of GLP‐2 [20]. Case reports have described the effects of DPP‐4 inhibitors (e.g., vildagliptin and sitagliptin) in patients with PPH [48, 49]. α‐synucleinopathies such as PD and DLB, which involve autonomic dysfunction, are characterized by intestinal α‐synuclein accumulation, highlighting the importance of gut–brain interactions [55]. GLP‐1 analogs have been reported to increase BP [56], and gut peptide–based therapies may represent a promising future direction for PPH management [21]. Recent studies have shown that exenatide improves off‐medication motor scores in PD [57] and that lixisenatide reduces motor disability progression at 12 months in early PD [58]. Further studies are warranted to evaluate the effects of GLP‐1 analogs on the autonomic function in patients with PPH associated with prodromal LBD. Additionally, in patients with type 2 diabetes and PPH, oral metformin has been shown to attenuate the hypotensive response to meals, potentially through the stimulation of GLP‐1 secretion and delayed gastric emptying [59].

### 2.7 Coexistence of Positional BP Dysregulation: OH and Supine Hypertension

In frail geriatric patients, 67% were found to have PPH, and 52% had OH; overall, 81% had either condition [5]. OH should be actively screened at the bedside in patients with PPH by measuring the BP and heart rate in the supine position and again after 3 min of standing. OH is diagnosed when SBP decreases by ≥ 20 mmHg and/or DBP by ≥ 10 mmHg from baseline. Standing SBP values of < 90 mmHg are also highly suggestive of OH and often predict orthostatic intolerance [51].

Patients with OH should receive careful education on symptom management [51], including:

1. Standing up slowly, especially after prolonged supine rest, and pausing in a seated position before rising.
2. Avoiding heat exposure, prolonged standing, alcohol, and large meals.
3. Adapting daily routines, such as showering while seated and voiding in a sitting position.
4. Performing BP‐raising maneuvers when dizziness occurs and sitting or lying down is not possible: for example, stepping in place, leg crossing, tensing the gluteal or abdominal muscles, bending forward, or clenching fists.

Supine hypertension is defined as SBP ≥ 140 mmHg and/or DBP ≥ 90 mmHg after > 5 min of supine rest [52]. Approximately half of the patients with OH develop neurogenic supine hypertension, which can be severe and persist for several hours during sleep [60]. Although typically asymptomatic, supine hypertension may interfere with effective OH treatment, exacerbate nocturnal pressure natriuresis, and increase the risk of hypertensive emergencies.

Non‐pharmacological management strategies for supine hypertension include:

1. Elevating the head of the bed by 10°–20° during sleep.
2. Consuming a small bedtime snack to induce PPH.
3. Providing nighttime enteral nutrition in gastrostomy‐fed patients.

Evaluating OH using ABPM is challenging owing to the inability to determine the exact timing of postural changes. However, exaggerated BP variability, defined as a daytime ambulatory BP standard deviation of > 16 mmHg, may indicate autonomic dysfunction [52, 61]. Patients with cardiovascular autonomic failure often exhibit nocturnal and supine hypertension, along with a loss of the physiological nocturnal BP dip (≥ 10%) while supine and asleep. This nondipping status may also be influenced by reduced renal function, salt sensitivity, and sleep apnea, which should be differentiated from true supine hypertension.

HBPM is recommended to gain further insights into circadian BP regulation. Although no validated protocol currently exists for HBPM in patients with cardiovascular autonomic failure, it is recommended that home BP be recorded three times daily–early morning, after lunch, and at bedtime–in the supine, seated, and standing positions for 1 week during the initial diagnostic work‐up [52].

## 3 Conclusion

PPH is observed in approximately half of the older adults and is often associated with autonomic dysfunction. Its occurrence is influenced by factors such as meal size, carbohydrate content, and time of day, particularly in the morning, likely due to postprandial splanchnic hyperemia and the effects of intestinal peptides. PPH is commonly defined as a fall in SBP of > 20 mmHg or a decrease to < 90 mmHg when the preprandial SBP exceeds 100 mmHg within 2 h after a meal. However, diagnostic protocols vary across studies in terms of test meal composition and BP measurement methods.

PPH can be evaluated using ABPM or HBPM, each with distinct advantages and limitations. Non‐pharmacological interventions remain the first‐line approach to management, with careful attention paid to coexisting OH and supine hypertension. Among the pharmacological options, acarbose, an α‐glucosidase inhibitor, has shown efficacy in mitigating PPH. Recently, clinical interest has grown in therapies targeting intestinal hormones, such as GLP‐1 and GLP‐2, which may offer promising avenues for future treatment.

## Author Contributions

Joji Ishikawa was responsible for the accuracy of the paper collection, concept and design, and manuscript drafting. All authors contributed in critical revisions.

## Funding

The corresponding author (J.I.) was supported in part by a research grant from Tokyo Metropolitan Institute for Geriatrics and Gerontology and research funding for longevity sciences (22‐9) from the National Center for Geriatrics and Gerontology (NCGG), Japan (J.I. and A.T.).

## Ethics Statement

The authors have nothing to report.

## Conflicts of Interest

The authors declare no conflicts of interest.

## Data Availability

Data sharing is not applicable to this article as no datasets were generated or analyzed during the current study.

## References

[1] L. Huang , S. Li , X. Xie , et al., “Prevalence of Postprandial Hypotension in Older Adults: A Systematic Review and Meta‐Analysis,” Age and Ageing 53 (2024): 1–9. 10.1093/ageing/afae022 PMC10898335 38411408

[2] X. Zhou , T. Wu , M. Sang , et al., “Variations in Blood Pressure After a 75 g Oral Glucose Load and Their Implications for Detecting Hypertension and Postprandial Hypotension in Chinese Adults: A Cross‐Sectional Study,” European Journal of Preventive Cardiology 32 (2025): 1382–1391. 40197834 10.1093/eurjpc/zwaf217

[3] A. Pavelić , M. Krbot Skorić , L. Crnošija , and M. Habek , “Postprandial Hypotension in Neurological Disorders: Systematic Review and Meta‐Analysis,” Clinical Autonomic Research 27 (2017): 263–271. 28647892 10.1007/s10286-017-0440-8

[4] P. E. Mehr , P. J. Ortiz , K. R. O'Rourke , et al., “Peripheral Autonomic Failure Is Associated With More Severe Postprandial Hypotension Compared to Central Autonomic Failure,” Clinical Autonomic Research 35 (2025): 607–616. 40360851 10.1007/s10286-025-01131-x PMC12325557

[5] L. C. Vloet , R. E. Pel‐Little , P. A. Jansen , and R. W. Jansen , “High Prevalence of Postprandial and Orthostatic Hypotension Among Geriatric Patients Admitted to Dutch Hospitals,” Journals of Gerontology. Series A, Biological Sciences and Medical Sciences 60 (2005): 1271–1277. 16282558 10.1093/gerona/60.10.1271

[6] R. W. Jansen and L. A. Lipsitz , “Postprandial Hypotension: Epidemiology, Pathophysiology, and Clinical Management,” Annals of Internal Medicine 122 (1995): 286–295. 7825766 10.7326/0003-4819-122-4-199502150-00009

[7] A. T. Isik , M. S. Ontan , F. S. Dost , et al., “Postprandial Hypotension Is More Common Than Orthostatic Hypotension in Older Adults With Dementia With Lewy Bodies: A Cross‐Sectional Study,” Hypertension Research 47 (2024): 2840–2846. 39138363 10.1038/s41440-024-01829-x PMC11456507

[8] R. W. Jansen , C. M. Connelly , M. M. Kelley‐Gagnon , J. A. Parker , and L. A. Lipsitz , “Postprandial Hypotension in Elderly Patients With Unexplained Syncope,” Archives of Internal Medicine 155 (1995): 945–952. 7726703

[9] K. Kohara , Y. Jiang , M. Igase , et al., “Postprandial Hypotension Is Associated With Asymptomatic Cerebrovascular Damage in Essential Hypertensive Patients,” Hypertension 33 (1999): 565–568. 9931166 10.1161/01.hyp.33.1.565

[10] Y. Tabara , Y. Okada , E. Uetani , et al., “Postprandial Hypotension as a Risk Marker for Asymptomatic Lacunar Infarction,” Journal of Hypertension 32 (2014): 1084–1090. 24695394 10.1097/HJH.0000000000000150

[11] R. W. Jansen , “Postprandial Hypotension: Simple Treatment but Difficulties With the Diagnosis,” Journals of Gerontology. Series A, Biological Sciences and Medical Sciences 60 (2005): 1268–1270. 16282557 10.1093/gerona/60.10.1268

[12] W. S. Aronow and C. Ahn , “Association of Postprandial Hypotension With Incidence of Falls, Syncope, Coronary Events, Stroke, and Total Mortality at 29‐Month Follow‐Up in 499 Older Nursing Home Residents,” Journal of the American Geriatrics Society 45 (1997): 1051–1053. 9288010 10.1111/j.1532-5415.1997.tb05965.x

[13] D. J. A. Jenkins , S. Sahye‐Pudaruth , K. Khodabandehlou , et al., “Systematic Review and Meta‐Analysis Examining the Relationship Between Postprandial Hypotension, Cardiovascular Events, and All‐Cause Mortality,” American Journal of Clinical Nutrition 116 (2022): 663–671. 35675216 10.1093/ajcn/nqac158 PMC9437988

[14] A. A. Fisher , M. W. Davis , W. Srikusalanukul , and M. M. Budge , “Postprandial Hypotension Predicts All‐Cause Mortality in Older, Low‐Level Care Residents,” Journal of the American Geriatrics Society 53 (2005): 1313–1320. 16078956 10.1111/j.1532-5415.2005.53415.x

[15] A. Jang , “Postprandial Hypotension as a Risk Factor for the Development of New Cardiovascular Disease: A Prospective Cohort Study With 36 Month Follow‐Up in Community‐Dwelling Elderly People,” Journal of Clinical Medicine 9 (2020): 9. 10.3390/jcm9020345 PMC7073667 32012696

[16] L. C. Vloet , D. J. Mehagnoul‐Schipper , W. H. Hoefnagels , and R. W. Jansen , “The Influence of Low‐, Normal‐, and High‐Carbohydrate Meals on Blood Pressure in Elderly Patients With Postprandial Hypotension,” Journals of Gerontology. Series A, Biological Sciences and Medical Sciences 56 (2001): M744–M748. 11723147 10.1093/gerona/56.12.m744

[17] W. Qiao , J. Li , Y. Li , et al., “Acarbose, the α‐Glucosidase Inhibitor, Attenuates the Blood Pressure and Splanchnic Blood Flow Responses to Meal in Elderly Patients With Postprandial Hypotension Concomitant With Abnormal Glucose Metabolism,” Blood Pressure Monitoring 21 (2016): 38–42. 26474001 10.1097/MBP.0000000000000160

[18] L. G. Trahair , M. Horowitz , and K. L. Jones , “Postprandial Hypotension Is Associated With More Rapid Gastric Emptying in Healthy Older Individuals,” Journal of the American Medical Directors Association 16 (2015): 521–523. 25769959 10.1016/j.jamda.2015.01.097

[19] H. Pham , L. Phillips , L. Trahair , S. Hatzinikolas , M. Horowitz , and K. L. Jones , “Longitudinal Changes in the Blood Pressure Responses to, and Gastric Emptying of, an Oral Glucose Load in Healthy Older Subjects,” Journals of Gerontology. Series A, Biological Sciences and Medical Sciences 75 (2020): 244–248. 30689778 10.1093/gerona/glz014

[20] T. Fukushima , M. Asahina , Y. Fujinuma , et al., “Role of Intestinal Peptides and the Autonomic Nervous System in Postprandial Hypotension in Patients With Multiple System Atrophy,” Journal of Neurology 260 (2013): 475–483. 22983428 10.1007/s00415-012-6660-x

[21] M. J. Borg , C. Xie , C. K. Rayner , M. Horowitz , K. L. Jones , and T. Wu , “Potential for Gut Peptide‐Based Therapy in Postprandial Hypotension,” Nutrients 13 (2021): 2826. 34444986 10.3390/nu13082826 PMC8399874

[22] O. Staneczek , N. Abbas‐Terki , F. Loew , and C. C. Sieber , “A Full Stomach but an Empty Head,” Journal of the American Geriatrics Society 49 (2001): 1262–1263. 11559395 10.1046/j.1532-5415.2001.49247.x

[23] K. M. Madden , B. Feldman , and G. S. Meneilly , “Baroreflex Function and Postprandial Hypotension in Older Adults,” Clinical Autonomic Research 31 (2021): 273–280. 32062813 10.1007/s10286-020-00671-8

[24] J. Lagro , A. Meel‐van den Abeelen , D. L. de Jong , B. W. Schalk , M. G. Olde Rikkert , and J. A. Claassen , “Geriatric Hypotensive Syndromes Are Not Explained by Cardiovascular Autonomic Dysfunction Alone,” Journals of Gerontology. Series A, Biological Sciences and Medical Sciences 68 (2013): 581–589. 23070881 10.1093/gerona/gls214

[25] H. Hu , W. Qiao , X. Wang , et al., “Effect of Blood Insulin Level on Postprandial Hypotension in Elderly People,” Blood Pressure Monitoring 25 (2020): 201–205. 32404599 10.1097/MBP.0000000000000450

[26] R. E. Karakaya , A. A. Tam , S. Fakı , et al., “Postprandial Hypotension in Adults: Exploring Insulin Dynamics During a Mixed Meal Test,” Nutrients 17 (2025): 479. 39940337 10.3390/nu17030479 PMC11821074

[27] R. Abbas , A. Tanguy , D. Bonnet‐Zamponi , R. Djedid , A. Lounis , and M. L. Gaubert‐Dahan , “New Simplified Screening Method for Postprandial Hypotension in Older People,” Journal of Frailty & Aging 7 (2018): 28–33. 29412439 10.14283/jfa.2018.2

[28] L. C. Vloet , R. Smits , and R. W. Jansen , “The Effect of Meals at Different Mealtimes on Blood Pressure and Symptoms in Geriatric Patients With Postprandial Hypotension,” Journals of Gerontology. Series A, Biological Sciences and Medical Sciences 58 (2003): 1031–1035. 14630885 10.1093/gerona/58.11.m1031

[29] F. Puisieux , H. Bulckaen , A. L. Fauchais , S. Drumez , F. Salomez‐Granier , and P. Dewailly , “Ambulatory Blood Pressure Monitoring and Postprandial Hypotension in Elderly Persons With Falls or Syncopes,” Journals of Gerontology. Series A, Biological Sciences and Medical Sciences 55 (2000): M535–M540. 10995052 10.1093/gerona/55.9.m535

[30] F. Puisieux , D. Court , E. Baheu , C. Dipompeo , H. Bulckaen , and P. Dewailly , “Intraindividual Reproducibility of Postprandial Hypotension,” Gerontology 48 (2002): 315–320. 12169798 10.1159/000065256

[31] R. W. Jansen , M. M. Kelly‐Gagnon , and L. A. Lipsitz , “Intraindividual Reproducibility of Postprandial and Orthostatic Blood Pressure Changes in Older Nursing‐Home Patients: Relationship With Chronic Use of Cardiovascular Medications,” Journal of the American Geriatrics Society 44 (1996): 383–389. 8636581 10.1111/j.1532-5415.1996.tb06406.x

[32] K. Kohara , K. Uemura , Y. Takata , T. Okura , Y. Kitami , and K. Hiwada , “Postprandial Hypotension: Evaluation by Ambulatory Blood Pressure Monitoring,” American Journal of Hypertension 11 (1998): 1358–1363. 9832180 10.1016/s0895-7061(98)00161-7

[33] L. G. Trahair , M. Horowitz , and K. L. Jones , “Postprandial Hypotension: A Systematic Review,” Journal of the American Medical Directors Association 15 (2014): 394–409. 24630686 10.1016/j.jamda.2014.01.011

[34] T. Grodzicki , M. Rajzer , R. Fagard , et al., “Ambulatory Blood Pressure Monitoring and Postprandial Hypotension in Elderly Patients With Isolated Systolic Hypertension. Systolic Hypertension in Europe (SYST‐EUR) Trial Investigators,” Journal of Human Hypertension 12 (1998): 161–165. 9579765 10.1038/sj.jhh.1000573

[35] A. Zanasi , E. Tincani , V. Evandri , P. Giovanardi , M. Bertolotti , and G. Rioli , “Meal‐Induced Blood Pressure Variation and Cardiovascular Mortality in Ambulatory Hypertensive Elderly Patients: Preliminary Results,” Journal of Hypertension 30 (2012): 2125–2132. 22929611 10.1097/HJH.0b013e328357f16d

[36] K. F. Alquadan , G. Singhania , A. Koratala , and A. A. Ejaz , “Office Orthostatic Blood Pressure Measurements and Ambulatory Blood Pressure Monitoring in the Prediction of Autonomic Dysfunction,” Clinical Hypertension 23 (2017): 3. 28331633 10.1186/s40885-016-0059-4 PMC5351249

[37] A. Kitae , E. Ushigome , Y. Hashimoto , et al., “Asymptomatic Postprandial Hypotension in Patients With Diabetes: The KAMOGAWA‐HBP Study,” Journal of Diabetes Investigation 12 (2021): 837–844. 33000524 10.1111/jdi.13418 PMC8089004

[38] J. Barochiner , J. Alfie , L. S. Aparicio , et al., “Postprandial Hypotension Detected Through Home Blood Pressure Monitoring: A Frequent Phenomenon in Elderly Hypertensive Patients,” Hypertension Research 37 (2014): 438–443. 24108236 10.1038/hr.2013.144

[39] J. Alfie , “Utility of Home Blood Pressure Monitoring to Evaluate Postprandial Blood Pressure in Treated Hypertensive Patients,” Therapeutic Advances in Cardiovascular Disease 9 (2015): 133–139. 26187907 10.1177/1753944715593444

[40] E. Sasaki , H. Kitaoka , and N. Ohsawa , “Postprandial Hypotension in Patients With Non‐Insulin‐Dependent Diabetes Mellitus,” Diabetes Research and Clinical Practice 18 (1992): 113–121. 1478151 10.1016/0168-8227(92)90007-e

[41] B. Grobéty , E. K. Grasser , G. Yepuri , A. G. Dulloo , and J. P. Montani , “Postprandial Hypotension in Older Adults: Can It Be Prevented by Drinking Water Before the Meal?,” Clinical Nutrition 34 (2015): 885–891. 25277381 10.1016/j.clnu.2014.09.009

[42] A. S. Oberman , R. K. Harada , M. M. Gagnon , D. K. Kiely , and L. A. Lipsitz , “Effects of Postprandial Walking Exercise on Meal‐Related Hypotension in Frail Elderly Patients,” American Journal of Cardiology 84 (1999): 1130–1132. 10569685 10.1016/s0002-9149(99)00520-2

[43] B. Wang , J. Zhao , Q. Zhan , et al., “Acarbose for Postprandial Hypotension With Glucose Metabolism Disorders: A Systematic Review and Meta‐Analysis,” Frontiers in Cardiovascular Medicine 8 (2021): 663635, 10.3389/fcvm.2021.663635. 34095252 PMC8172613

[44] C. Shibao , A. Gamboa , A. Diedrich , et al., “Acarbose, an α‐Glucosidase Inhibitor, Attenuates Postprandial Hypotension in Autonomic Failure,” Hypertension 50 (2007): 54–61. 17515447 10.1161/HYPERTENSIONAHA.107.091355

[45] T. Maruta , K. Komai , M. Takamori , and M. Yamada , “Voglibose Inhibits Postprandial Hypotension in Neurologic Disorders and Elderly People,” Neurology 66 (2006): 1432–1434. 16682681 10.1212/01.wnl.0000214102.65215.76

[46] A. Awosika , U. Adabanya , R. M. Millis , A. E. Omole , and J. H. Moon , “Postprandial Hypotension: An Underreported Silent Killer in the Aged,” Cureus 15 (2023): e35411. 36851946 10.7759/cureus.35411 PMC9964048

[47] K. Furukawa , T. Suzuki , H. Ishiguro , et al., “Prevention of Postprandial Hypotension‐Related Syncope by Caffeine in a Patient With Long‐Standing Diabetes Mellitus,” Endocrine Journal 67 (2020): 585–592. 32115439 10.1507/endocrj.EJ19-0370

[48] A. Yonenaga , H. Ota , M. Honda , et al., “Marked Improvement of Elderly Postprandial Hypotension by Dipeptidyl Peptidase IV Inhibitor,” Geriatrics & Gerontology International 13 (2013): 227–229. 23286563 10.1111/j.1447-0594.2012.00903.x

[49] Y. Saito , J. Ishikawa , and K. Harada , “Postprandial and Orthostatic Hypotension Treated by Sitagliptin in a Patient With Dementia With Lewy Bodies,” American Journal of Case Reports 17 (2016): 887–893. 27885251 10.12659/AJCR.900620 PMC5127632

[50] J. W. Park , L. E. Okamoto , C. A. Shibao , and I. Biaggioni , “Pharmacologic Treatment of Orthostatic Hypotension,” Autonomic Neuroscience 229 (2020): 102721. 32979782 10.1016/j.autneu.2020.102721 PMC7704612

[51] A. Fanciulli , F. Leys , C. Falup‐Pecurariu , R. Thijs , and G. K. Wenning , “Management of Orthostatic Hypotension in Parkinson's Disease,” Journal of Parkinson's Disease 10 (2020): S57–S64. 10.3233/JPD-202036 PMC7592655 32716319

[52] A. Fanciulli , J. Jordan , I. Biaggioni , et al., “Consensus Statement on the Definition of Neurogenic Supine Hypertension in Cardiovascular Autonomic Failure by the American Autonomic Society (AAS) and the European Federation of Autonomic Societies (EFAS): Endorsed by the European Academy of Neurology (EAN) and the European Society of Hypertension (ESH),” Clinical Autonomic Research 28 (2018): 355–362. 29766366 10.1007/s10286-018-0529-8 PMC6097730

[53] D. J. Mehagnoul‐Schipper , R. H. Boerman , W. H. Hoefnagels , and R. W. Jansen , “Effect of Levodopa on Orthostatic and Postprandial Hypotension in Elderly Parkinsonian Patients,” Journals of Gerontology. Series A, Biological Sciences and Medical Sciences 56 (2001): M749–M755. 11723148 10.1093/gerona/56.12.m749

[54] J. Zhang and L. Guo , “Effectiveness of Acarbose in Treating Elderly Patients With Diabetes With Postprandial Hypotension,” Journal of Investigative Medicine 65 (2017): 772–783. 28213385 10.1136/jim-2016-000295

[55] E. Menozzi , A. H. V. Schapira , and P. Borghammer , “The Gut‐Brain Axis in Parkinson Disease: Emerging Concepts and Therapeutic Implications,” Movement Disorders Clinical Practice 12 (2025): 904–916. 40079755 10.1002/mdc3.70029 PMC12275011

[56] H. Yamamoto , C. E. Lee , J. N. Marcus , et al., “Glucagon‐Like Peptide‐1 Receptor Stimulation Increases Blood Pressure and Heart Rate and Activates Autonomic Regulatory Neurons,” Journal of Clinical Investigation 110 (2002): 43–52. 12093887 10.1172/JCI15595 PMC151031

[57] D. Athauda , K. Maclagan , S. S. Skene , et al., “Exenatide Once Weekly Versus Placebo in Parkinson's Disease: A Randomised, Double‐Blind, Placebo‐Controlled Trial,” Lancet 390 (2017): 1664–1675. 28781108 10.1016/S0140-6736(17)31585-4 PMC5831666

[58] W. G. Meissner , P. Remy , C. Giordana , et al., “Trial of Lixisenatide in Early Parkinson's Disease,” New England Journal of Medicine 390 (2024): 1176–1185. 38598572 10.1056/NEJMoa2312323

[59] D. R. Quast , C. Xie , M. J. Bound , et al., “Effects of Metformin on Postprandial Blood Pressure, Heart Rate, Gastric Emptying, GLP‐1, and Prevalence of Postprandial Hypotension in Type 2 Diabetes: A Double‐Blind Placebo‐Controlled Crossover Study,” Diabetes 74 (2025): 611–618, 10.2337/db24-0830. 39761379

[60] A. Fanciulli , G. Göbel , J. P. Ndayisaba , et al., “Supine Hypertension in Parkinson's Disease and Multiple System Atrophy,” Clinical Autonomic Research 26 (2016): 97–105. 26801189 10.1007/s10286-015-0336-4

[61] H. A. Lodhi , P. A. Peri‐Okonny , K. Schesing , et al., “Usefulness of Blood Pressure Variability Indices Derived From 24‐Hour Ambulatory Blood Pressure Monitoring in Detecting Autonomic Failure,” Journal of the American Heart Association 8 (2019): e010161. 30905258 10.1161/JAHA.118.010161 PMC6509738

